# Chronic jet lag alters gut microbiome and mycobiome and promotes the progression of MAFLD in HFHFD-fed mice

**DOI:** 10.3389/fmicb.2023.1295869

**Published:** 2023-12-07

**Authors:** Ruoyi Zheng, Xingwei Xiang, Ying Shi, Anqi Qiu, Xin Luo, Junyan Xie, Ryan Russell, Dongmei Zhang

**Affiliations:** ^1^Department of Endocrinology, Xiangya Hospital, Central South University, Changsha, Hunan, China; ^2^Key Laboratory of Diabetes Immunology (Central South University), Ministry of Education, Changsha, China; ^3^Department of Health and Human Performance, College of Health Professions, University of Texas Rio Grande Valley, Brownsville, TX, United States; ^4^Hunan Engineering Research Center for Obesity and its Metabolic Complications, Xiangya Hospital, Central South University, Changsha, China

**Keywords:** circadian disruption, chronic jet lag, microbiome, metabolic-dysfunction associated fatty liver disease, mycobiome

## Abstract

Metabolic dysfunction-associated fatty liver disease (MAFLD) is the most common chronic liver disease worldwide. Circadian disruptors, such as chronic jet lag (CJ), may be new risk factors for MAFLD development. However, the roles of CJ on MAFLD are insufficiently understood, with mechanisms remaining elusive. Studies suggest a link between gut microbiome dysbiosis and MAFLD, but most of the studies are mainly focused on gut bacteria, ignoring other components of gut microbes, such as gut fungi (mycobiome), and few studies have addressed the rhythm of the gut fungi. This study explored the effects of CJ on MAFLD and its related microbiotic and mycobiotic mechanisms in mice fed a high fat and high fructose diet (HFHFD). Forty-eight C57BL6J male mice were divided into four groups: mice on a normal diet exposed to a normal circadian cycle (ND-NC), mice on a normal diet subjected to CJ (ND-CJ), mice on a HFHFD exposed to a normal circadian cycle (HFHFD-NC), and mice on a HFHFD subjected to CJ (HFHFD-CJ). After 16 weeks, the composition and rhythm of microbiota and mycobiome in colon contents were compared among groups. The results showed that CJ exacerbated hepatic steatohepatitis in the HFHFD-fed mice. Compared with HFHFD-NC mice, HFHFD-CJ mice had increases in *Aspergillus*, *Blumeria* and lower abundances of *Akkermansia*, *Lactococcus*, *Prevotella*, *Clostridium*, *Bifidobacterium*, *Wickerhamomyces*, and *Saccharomycopsis* genera. The fungi-bacterial interaction network became more complex after HFHFD and/or CJ interventions. The study revealed that CJ altered the composition and structure of the gut bacteria and fungi, disrupted the rhythmic oscillation of the gut microbiota and mycobiome, affected interactions among the gut microbiome, and promoted the progression of MAFLD in HFHFD mice.

## 1 Introduction

With a 25% global prevalence rate, metabolic dysfunction-associated fatty liver disease (MAFLD), formerly known as nonalcoholic fatty liver disease (NAFLD), is becoming the most common chronic liver disease worldwide (Younossi et al., 2018; Eslam et al., 2020). The majority of patients with MAFLD remain asymptomatic, although approximately 20–30% progress to develop chronic hepatic inflammation and advanced liver disease (fibrosis, cirrhosis, and hepatocellular carcinoma), and increased mortality.

Although the mechanisms responsible for the progression of MAFLD and its underlying pathogenesis remain unclear, environmental components appear to mediate the disease process. Among the environmental factors, circadian clock disruption has gained increasing interest in recent years. The circadian clock is an endogenous timekeeper system that controls and optimizes biological processes (Buijs et al., 2016). Many metabolic processes demonstrate circadian rhythm and are under the control of the circadian clock (Panda, 2016), thus circadian clock disruption may result in negative consequences for metabolic homeostasis. Evidence suggests that circadian disruption is involved in MAFLD (Mukherji et al., 2020; Saran et al., 2020). The two most common causes of circadian disruption in today’s 7/24 society are jet lag and shift work, and jet lag is considered analogous to shift work (Walker et al., 2020; Faraut et al., 2022), but few controlled studies have addressed the effects of chronic jet lag (CJ) on MAFLD, and its mechanisms remain incompletely understood.

A link between the gut microbiome and MAFLD has been previously suggested (Zhu et al., 2014; Safari and Gérard, 2019). Clinical studies and animal experiments have shown that gut microbiome dysbiosis contributes to the pathogenesis of MAFLD and its associated metabolic diseases (Le Roy et al., 2013; Aron-Wisnewsky et al., 2020a). Also, studies have demonstrated that the gut microbiota exhibits rhythmicity in composition and function, producing oscillations in key metabolic mediators that can be integrated into host circadian rhythms to maintain metabolic homeostasis (Bishehsari et al., 2020; Murakami and Tognini, 2020; Wang et al., 2022). Yet, the influences of circadian disruption on gut microbiota are not clear.

In addition, most studies have mainly focused on gut bacteria, ignoring other components of gut microbes, such as gut fungi. Gut commensal fungi, collectively referred to as the mycobiome, comprise a very small portion of gut microbes, but crosstalk with gut bacteria in many ways regarding growth, nutrition, reproduction, and pathogenicity (Zhang et al., 2022; Maas et al., 2023). Unlike bacteria, fungi are eukaryotes with complex cell structures. They can use more complex biologic processes and produce metabolites that can remain in an organism and cause damage even after they have been eradicated (Fotis et al., 2022; Zeng and Schnabl, 2022). There is agreement that a balanced gut mycobiome contributes to the maintenance of the homeostasis of the gut bacterial composition and host mucosal immune responses (van Tilburg Bernardes et al., 2020; Doron et al., 2021). Changes in fecal fungi have been described in several pathological conditions, and the contributions of the gut mycobiome to liver diseases are increasingly recognized (Kapitan et al., 2019). Recently, Demir et al. reported that non-obese MAFLD patients with advanced disease stages have a distinct composition of fecal fungi and display an increased systemic immune response to *Candida albicans*, and antifungals alleviate liver damage in mice that received fecal microbial transplants from patients with steatohepatitis (Demir et al., 2022). Mbaye et al. found higher occurrences of fungi. *Pichia kudriavzevii*, *Candida glabrata, Candida albicans,* and *Galactomyces geotrichum,* as well as increased levels of fecal alcohol in patients with steatohepatitis. Further *in vitro* analysis revealed that *Pichia kudriavzevii* involves in triglyceride production, and *Pichia kudriavzevii*, *Candida glabrata*, and *Candida albicans* tend to produce more ethanol from fructose (Mbaye et al., 2022). These findings indicate the pathogenic roles of the gut mycobiome in the progression of MAFLD. Nevertheless, research on the gut mycobiome is still in its infancy.

In the present study, we established a model in which mice were subjected to periodic changes in the light–dark cycle to mimic CJ conditions. We aimed to observe the influence of circadian disruption caused by CJ on the development of MAFLD in a high-fat and high-fructose diet (HFHFD)-induced MAFLD mice model, and to explore if the effects on MAFLD were associated with changes in the microbiome and mycobiome in colon contents.

## 2 Materials and methods

### 2.1 Animal study

**Figure 1:**
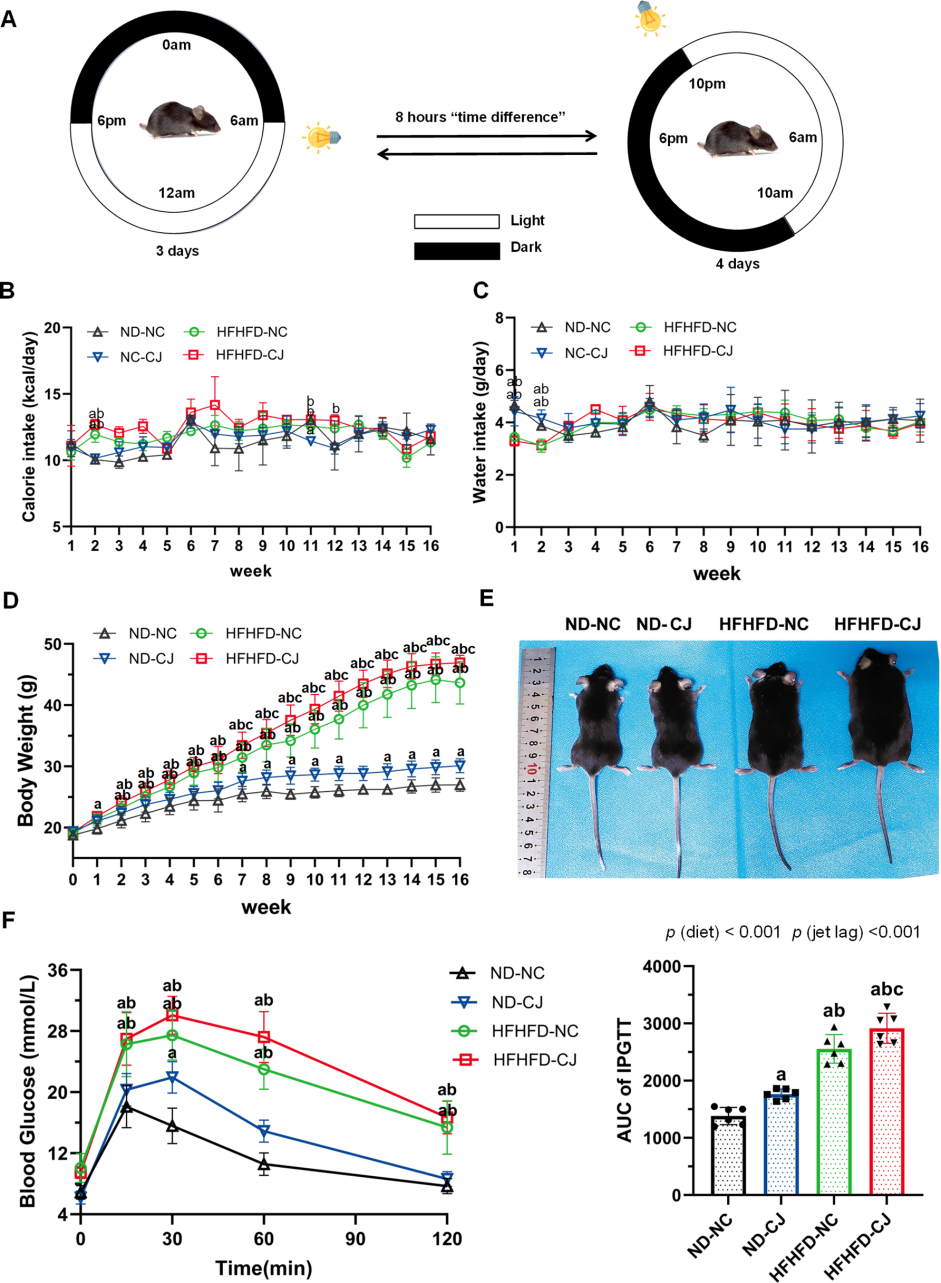
Effects of chronic jet lag on body weight and glucose homeostasis in HFHFD-fed mice. **(A)** Schematic representation of the jet lag model. The light phase is represented in white; the dark phase is represented in black. **(B)** Calorie intake, **(C)** water intake, **(D)** body weight, and **(E)** representative photographs of mice in each group. **(F)** IPGTT and AUC of IPGTT at 16th week. Differences were determined using either a one-way ANOVA **(B–D)** or a two-way ANOVA **(F)** followed by a Tukey’s multiple comparisons test. Data were expressed as Mean ± SD (*n* = 12 for **B–D**, *n* = 6 for **F**).^a^*p* < 0.05, vs. ND-NC group; ^b^*p* < 0.05, vs. ND-CJ group; ^c^*p* < 0.05, vs. HFHFD-NC group. *p* (diet), main effect of diet; *p* (jet leg), main effect of jet leg; *p* (light × jet leg), interaction effect of jet leg and diet.

Forty-eight male C57BL6J mice (5-week-old, 18–20 g) were purchased from the Hunan Slac-Jingda Laboratory Animal Co. (Changsha, China). After one week of acclimation, mice were randomly divided into four groups (n = 12 per group) as follow: ① mice were fed a normal diet (ND) and exposed to a normal lighting condition (ND-NC) (ND, fat 12%, carbohydrate 66%, protein 22%, 3.50 kcal/g), ② mice were fed an ND and underwent experimental CJ (ND-CJ), ③mice on an HFHFD and exposed to a normal lighting condition (HFHFD-NC), HFHFD is composed of high-fat diet (Research Diets-USA, D12492, fat 60%, carbohydrate 20%, protein 20%, 5.40 kcal/g) and high fructose (10% g/v) in the drinking water (Park et al., 2020), and ④mice on an HFHFD underwent experimental CJ (HFHFD-CJ). All the mice were housed in one room, and were housed in cages with four individuals per cage, and were provided with *ad libitum* access to water and food under controlled conditions (22 ± 2°C, 40 ~ 50% humidity). The HFHFD group mice received fructose water as the only drinking water. For the normal lighting condition, the mice were kept under strict light–dark cycles with lights on at 6 AM and off at 6 PM. For CJ, mice were housed for three days per week under normal light conditions and for the remaining four days of the week under an 8-h time difference (lights on at 10 PM and off at 10 AM) (Figure 1A). Experiments performed on CJ mice were performed when the mice were in the same light–dark cycle as control mice, and zeitgeber times (ZTs: the time when the lights were on) were synchronized (i.e., ZT0 of CJ mice corresponded to ZT0 of control mice). Twenty-four-hour food intake and body weight were recorded weekly for all animals throughout the experiment for 16 weeks. Animal protocols were approved by the Animal Use and Care Committee of Central South University and were conducted according to the regulations set by Central South University (No.2020sydw0989).

### 2.2 Intraperitoneal glucose tolerance test (IPGTT)

The IPGTT was performed at the beginning of the 16th week after a 5-h fast in the afternoon from 3:00 PM to 5:30 PM. Briefly, the mice were weighed and kept in a separate cage, then the mice were given an intraperitoneal injection of 50% D-glucose (2.0 g/kg), blood glucose levels were measured through tail vein sampling at 0, 15, 30, 60, and 120 min after glucose injection using a portable glucose monitor (ACCU-CHEK; Roche Diagnostics, Mannheim, Germany).

### 2.3 Body composition measurements

Dual Energy X-ray Absorptiometry (DXA, GE Lunar Corp., United States) was used to evaluate body fat rate using small animal software (GE Medical Systems Lunar, Madison, WI, United States).

### 2.4 Sample collection

After 16 weeks of intervention, mice were sacrificed at 6 h intervals on two consecutive days after fasting overnight, with ZT2 (8 AM) standing for morning, ZT8 (2 PM) for afternoon, ZT14 (8 PM) for evening, and ZT20 (2 AM) for night. Blood was collected from the retro-orbital vein after the mice were anesthetized (1% sodium pentobarbital solution, 50 mg/kg, injected intraperitoneally) and centrifuged at 3000 rpm for 10 min to isolate serum (stored at −20°C). Liver tissues and epididymal fat pads (visceral fat) were collected and weighed. Liver tissues were then either fixed with 4% paraformaldehyde solution or frozen in liquid nitrogen and stored at −80°C until analysis. Colon content samples were collected under a sterile fume to prevent miscellaneous bacterial contamination, frozen in liquid nitrogen, and stored at −80°C.

### 2.5 Liver pathology

Paraffin-embedded liver sections (4 μm) were stained with hematoxylin and eosin (H&E) or Masson’s trichrome stain. The NAFLD activity score (NAS) was assessed by an experienced physiologist using indices of inflammation, steatosis, and hepatocyte ballooning as previously described (Kleiner et al., 2005).

### 2.6 Serum analysis

Serum triglyceride (TG), total cholesterol (TC), low-density lipoprotein cholesterol (LDL-C), high-density lipoprotein cholesterol (HDL-C), alanine aminotransferase (ALT), and aspartate aminotransferase (AST) levels were measured using commercial reagents (Serotec Co, Sapporo, Japan) according to the manufacturer’s recommendations. Serum IL-6, IL-1β and TNF-α were measured using a commercial microspecific enzyme-linked immunosorbent assay (ELISA) kit (Cusabio, Wuhan, China).

### 2.7 Quantitative real time-PCR

Liver mRNA expression of circadian genes (*Clock, Bmal1, Cry1/2, Per1/2*) was assessed by quantitative real-time PCR. Primer sequences are listed in Supplementary Table S1. Gene expression was quantified using the ^△△^Ct method with glyceraldehyde 3 - phosphate dehydrogenase (GADPH) as an internal control.

### 2.8 Gut microbiome and mycobiome analysis

The total genomic DNA was extracted from the samples using the CTAB method. DNA concentration and purity were monitored using 1% agarose gels. According to the concentration, DNA was diluted to 1 ng/μL using sterile water. 16S rRNA genes of distinct regions (16S V3-V4) and ITS rRNA genes of distinct regions (ITS 1-5F) were amplified using specific primers (16S: 341F(5’-CCTAYGGGRBG CASCAG-3′), 806R(5’-GGACTACNNGGGTATCT AAT-3′), ITS: ITS5-1737F (5’-GGAAGTAAAAGTCGTAACAAGG-3′), ITS2-2043R (5’-GCTGCGTTCTTCATCGATGC-3′)) with the barcode. All PCR were carried out with 15 μL of Phusion® High-Fidelity PCR Master Mix (New England Biolabs). The same volume of 1 × TAE buffer was mixed with PCR products and electrophoresed on a 2% agarose gel for detection. The PCR products were mixed in equidensity ratios. The mixture of PCR products was purified using a Qiagen Gel Extraction Kit (Qiagen, Germany). Sequencing libraries were generated using the TruSeq® DNA PCR-Free Sample Preparation Kit (Illumina, United States), following the manufacturer’s recommendations, and index codes were added. Library quality was assessed on a Qubit@ 2.0 Fluorometer (Thermo Scientific). Finally, the library was sequenced on the Illumina NovaSeq platform and 250 bp paired-end reads were generated.

Raw data FASTQ files were imported into the format that could be operated by QIIME2 system using qiime tools import program. Demultiplexed sequences from each sample were quality filtered and trimmed, de-noised, merged, and then the chimeric sequences were identified and removed using the QIIME2 dada2 plugin to obtain the feature table of amplicon sequence variant (ASV). The QIIME2 feature-classifier plugin was then used to align the ASV sequences with the pre-trained GREENGENES 13_8 99% database and UNITE database (version 8.3) for bacteria and fungi, respectively. Sequence data analyses were mainly performed using QIIME and R (v.4.2.1) (RR Core Team, 2022). Alpha diversity indices, including Chao1, Shannon, and Observed features were calculated using the core-diversity plugin within QIIME2. PLS-DA was introduced as a supervised model to reveal the microbiota variation among groups using the R package “mixOmics (Rohart et al., 2017).” Analysis of variance (ANOVA) and LEfSe were employed to identify bacteria or fungi with different abundances among samples and groups using the python package (version 1.0.8). Correlation analysis was performed to reveal the association between microbial communities and environmental factors using the R package “vegan (Oksanen et al., 2022).” In addition, microbial functions were predicted by the phylogenetic investigation of communities by reconstructing unobserved states (PICRUSt) based on high-quality sequences. The Kyoto Encyclopedia of Genes and Genomes (KEGG) database was utilized to predict functional profiles for bacteria, and the MetaCyc database was used to predict functional profiles for fungi. The rhythmicity of the gut microbiota was assessed using the R package “cosinor2 (Mutak, 2018),” and *p* < 0.05 indicated a significant rhythm. Correlations between the ASVs were calculated using the R package “pheatmap (Kolde, 2019),” and significant correlations (adjusted *value of p* <0.05) were incorporated in network analysis. Network-level topological features were quantitatively assessed by calculating a set of metrics using the Gephi platform (version 0.9.2). And the Gephi platform was used for network visualization.

### 2.9 Statistical analysis

Statistical analyses were performed using SPSS (version 26.0; IBM, Armonk, United States) and GraphPad Prism version 8.0 (GraphPad Software Inc., San Diego, California, United States). Differences in changes of body weight, water intake, and caloric intake among groups were analyzed by a one-way ANOVA followed by a Tukey’s multiple comparisons test. Differences of the final outcome measures among groups were evaluated by a two-way ANOVA followed by a Tukey’s multiple comparison test. Data are shown as mean ± SD or mean ± SEM. Statistical significance was defined as *p* < 0.05. The statistical tests and sample sizes are indicated in the figure legends.

## 3 Results

### 3.1 CJ aggravated obesity and visceral adiposity in HFHFD-fed mice

Body weight in the ND-CJ group was significantly higher than that in the ND-NC group at the 7-11th week and after the 13th week (*p* < 0.05) (Figure 1D), although there were no significant differences in energy intake or water intake between the ND-NC and ND-CJ groups (Figures 1B,C). In HFHFD-fed mice, the body weight of HFHFD-CJ group was significantly higher than that of HFHFD-NC group after the 7th week (*p* < 0.05) (Figure 1D), while no differences in caloric intake were noted (Figure 1B). Representative photographs of mice in each group are shown in Figure 1E.

At the end of the experiment, HFHFD-fed mice displayed an increased body weight, liver weight, visceral fat weight, and body fat rate (fat %) than ND-fed mice (Table 1). In ND-fed mice, the body weight and fat % in the ND-CJ group were significantly increased than those in the ND-NC group. Compared to the HFHFD-NC group, the body weight, liver weight, liver %, and fat % were significantly increased in the HFHFD-CJ group (all *p* < 0.05) (Table 1).

**Table 1:** Characteristics of mice in each group at the end of experiment.

Parameters	Group ND-CJ HFHFD-NC HFHFD-CJ				2-Way ANOVA Statistics
	ND-NC	ND-CJ	HFHFD-NC	HFHFD-CJ	
BW(g)	26.5 ± 0.76	30.95 ± 1.16^a^	41.91 ± 4.45^a,b^	46.73 ± 1.48^a,b,c^	*p* (diet) <0.001 *p* (jet leg) <0.001
Liver (g)	1.15 ± 0.14	1.22 ± 0.13	1.68 ± 0.40^a,b^	1.98 ± 0.27^a,b,c^	*p* (diet) <0.001 *p* (jet leg) <0.05
Liver %	4.03 ± 0.24	4.23 ± 0.33	3.67 ± 0.52^b^	4.42 ± 0.51^c^	*p* (jet leg) <0.05
Visceral Fat (g)	0.33 ± 0.08	0.46 ± 0.016	2.19 ± 0.33^a,b^	2.20 ± 0.24^a,b^	*p* (diet) <0.001
Fat %	9.58 ± 1.80	15.90 ± 3.76^a^	50.38 ± 4.66^a,b^	56.52 ± 3.86^a,b,c^	*p* (diet) <0.001 *p* (jet leg) <0.001
TG (mmol/L)	1.17 ± 0.44	1.23 ± 0.32	1.28 ± 0.50	1.34 ± 0.33	ns
TC (mmol/L)	2.53 ± 0.25	2.68 ± 0.29	4.72 ± 0.83^a,b^	4.93 ± 0.43^a,b^	*p* (diet) <0.001
HDL-C (mmol/L)	1.67 ± 0.14	1.74 ± 0.14	3.07 ± 0.55^a,b^	3.17 ± 0.26^a,b^	*p* (diet) <0.001
LDL-C(mmol/L)	0.69 ± 0.18	0.71 ± 0.21	1.02 ± 0.20^a,b^	1.04 ± 0.11^a,b^	*p* (diet) <0.001
IL-6 (pg/mL)	4.37 ± 0.62	4.68 ± 0.96	4.77 ± 0.46	8.64 ± 1.43^a,b,c^	*p* (diet) <0.01 *p* (jet leg) <0.001 *p* (diet × light) < 0.01
IL-1β (pg/mL)	132.10 ± 25.96	189.00 ± 30.84	298.2 ± 62.16	288.7 ± 42.40	ns
TNF-α (pg/mL)	77.06 ± 1.94	92.47 ± 38.17	104.3 ± 11.79	365.0 ± 63.61^a,b,c^	*p* (diet) <0.01 *p* (jet leg) <0.05
FBG (mmol/L)	6.35 ± 1.75	7.69 ± 0.96	9.18 ± 1.74^a^	10.61 ± 2.83^a,b^	*p* (diet) <0.001 *p* (jet leg) <0.05

Data were expressed as Mean ± SD. Differences were determined using a two-way ANOVA followed by a Tukey’s multiple comparisons test. ^a^*p* < 0.05, vs. ND-NC group; ^b^*p* < 0.05, vs. ND-CJ group; ^c^*p* < 0.05, vs. HFHFD-NC group. *p* (diet), main effect of diet; *p* (jet leg), main effect of jet leg; *p* (light × jet leg), interaction effect of jet leg and diet. BW, body weight; liver %, liver weight to body weight ratio; fat %, body fat rate; TG, triglyceride; TC, total cholesterol; LDL-C, low-density lipoprotein cholesterol; HDL-C, high-density lipoprotein cholesterol; IL-6, interleukin-6; IL-1β, interleukin-1β; TNF-α, tumor necrosis factor α; FBG, fasting blood glucose.

### 3.2 Effects of CJ on serum lipid profiles, inflammatory cytokines, and glucose homeostasis

There were no significant differences in serum triglycerides (TG) levels among groups, while serum total cholesterol (TC), low-density lipoprotein cholesterol (LDL-C), and high-density lipoprotein cholesterol (HDL-C) levels were significantly higher in HFHFD-fed mice than in ND-fed mice [all *p* (diet) <0.001] (Table 1). Serum IL-6 and TNF-α levels were significantly elevated in the HFHFD-CJ group vs. the ND-NC, ND-CJ, and HFHFD-NC groups (Table 1). Compared to the ND-NC group, the HFHFD-CJ group and HFHFD-NC group had higher FBG [*p* (diet) <0.001, *p* (jet lag) <0.05] (Table 1).

Intraperitoneal glucose tolerance test (IPGTT) demonstrated that the blood glucose levels at 15, 30, 60, and 120 min were significantly elevated in HFHFD mice compared to ND mice (all *p* < 0.05) (Figure 1F). Compared to ND-NC mice, ND-CJ mice had higher blood glucose levels at 30 min during IPGTT (*p* < 0.05) and increased AUC for IPGTT (Figure 1F). The AUC for IPGTT in the HFHFD-CJ group was higher than those in the ND-NC, ND-CJ, and HFHFD-NC groups, with the significant main effects of both diet (*p* < 0.001) and jet lag (*p* < 0.001) (Figure 1F).

### 3.3 CJ promoted the progression of MAFLD in HFHFD-fed mice and altered liver expressions of core clock genes

**Figure 2:**
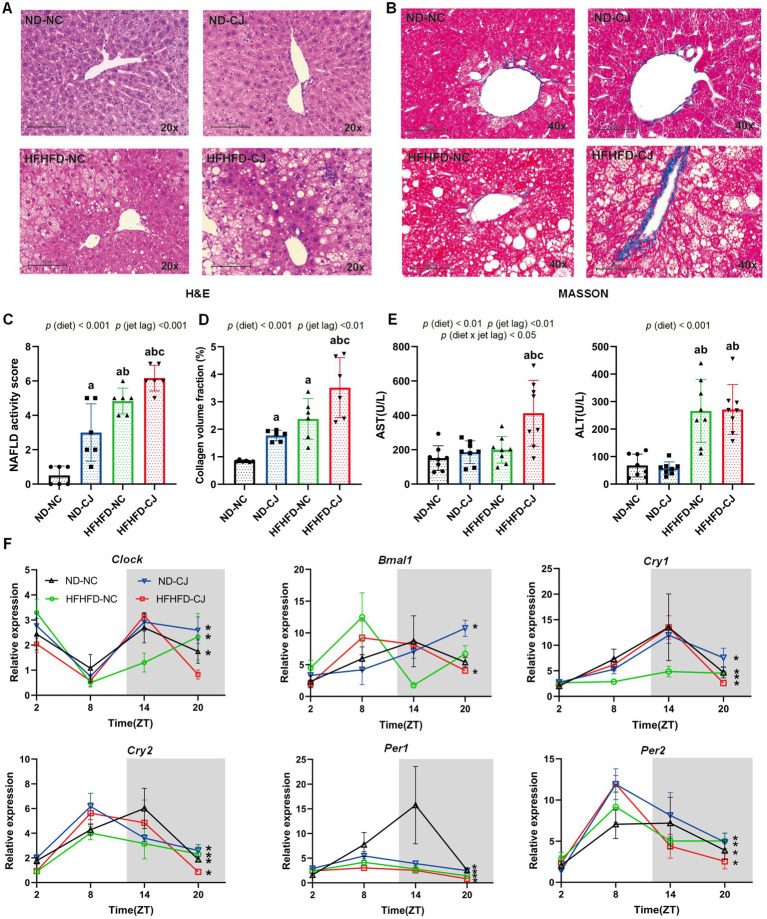
Chronic jet lag promoted the progression of MAFLD in HFHFD-fed mice and altered core clock gene expression in the liver. **(A)** Representative pictures of H&E staining (20 × magnifications) in liver tissue, **(B)** Representative pictures of MASSON staining (40× magnifications) in liver tissue, **(C)** NAFLD activity score, **(D)** the collagen volume fraction of MASSON staining. **(E)** Serum AST and ALS level in each group. **(F)** *Clock*, *Bmal1*, *Cry1*, *Cry2*, *Per1*, and *Per2* mRNA expression in the liver. Data were expressed as Mean ± SD **(C–E)** or Mean ± SEM **(F)**, n = 6 for **C,D**, n = 8 for **E**, *n* = 3 at each time point for **F**. Differences were determined using a two-way ANOVA followed by a Tukey’s multiple comparisons test, *^a^p* < 0.05, vs. ND-NC group; *^b^p* < 0.05, vs. ND-CJ group; *^c^p* < 0.05, vs. HFHFD-NC group. *p* (diet), main effect of diet; *p* (jet leg), main effect of jet leg; *p* (light × jet leg), interaction effect of jet leg and diet. AST, aspartate aminotransferase; ALT, alanine aminotransferase. Asterisks indicate the mRNA that exhibit significant oscillatory rhythmicity using the *Cosinor* analysis with a *value of p* of less than 0.05.

Histopathologically, mild steatosis and inflammatory cell infiltration were observed in the liver tissue of the ND-CJ group. In HFHFD-fed mice, hepatic steatosis and inflammatory infiltration were significantly elevated in the HFHFD-CJ group, and the HFHFD-CJ group had increased NAFLD activity scores (NAS) in comparison to the HFHFD-NC group [*p* (diet) <0.001, *p* (jet lag) <0.001] (Figures 2A,C). MASSON staining demonstrated a significant increase in collagen accumulation in the HFHFD-CJ group, and the collagen volume fraction was significantly higher in the HFHFD-CJ group vs. the HFHFD-NC group [*p* (diet) <0.001, *p* (jet lag) <0.01] (Figures 2B,D). Serum alanine aminotransferase (ALT) was significantly higher in HFHFD-fed mice vs. ND-fed mice [*p* (diet) <0.001], and serum aspartate aminotransferase (AST) was higher in the HFHFD-CJ group vs. the HFHFD-NC group [*p* (diet) <0.01, *p* (jet lag) <0.01, *p* (diet × jet lag) <0.05] (Figure 2E).

We further analysed the diurnal variation in core clock genes in liver tissues (Figure 2F). *Cry1*, *Cry2*, *Per1*, and *Per2* showed a daily rhythm in all groups. *Clock* mRNA expression showed diurnal rhythmicity in the livers of ND-NC, ND-CJ, and HFHFD-NC mice, but not in HFHFD-CJ mice, whereas *Bmal1* mRNA expression showed significant rhythmicity in ND-CJ and HFHFD-CJ mice, but not in ND-NC and HFHFD-NC mice. These mRNA expressions peaked at night in the ND-NC group. However, both *Cry2* and *Per1* mRNA expression peaked in the afternoon in the ND-CJ group. Compared to the ND-NC group, the ND-CJ group demonstrated a reduced amplitude of *Per1* mRNA expression and an increased amplitude of *Per2* mRNA expression. In the HFHFD-CJ group, *Bmal1, Cry2, Per1,* and *Per2* mRNA expression all peaked in the afternoon. Compared to the ND-NC group, the HFHFD-CJ group exhibited an increase in the amplitude of *Clock*, *Cry1*, and *Per2* mRNA expression, while the amplitude of *Per1* mRNA expression was reduced.

### 3.4 Changes in the composition of colon microbiota in mice

**Figure 3:**
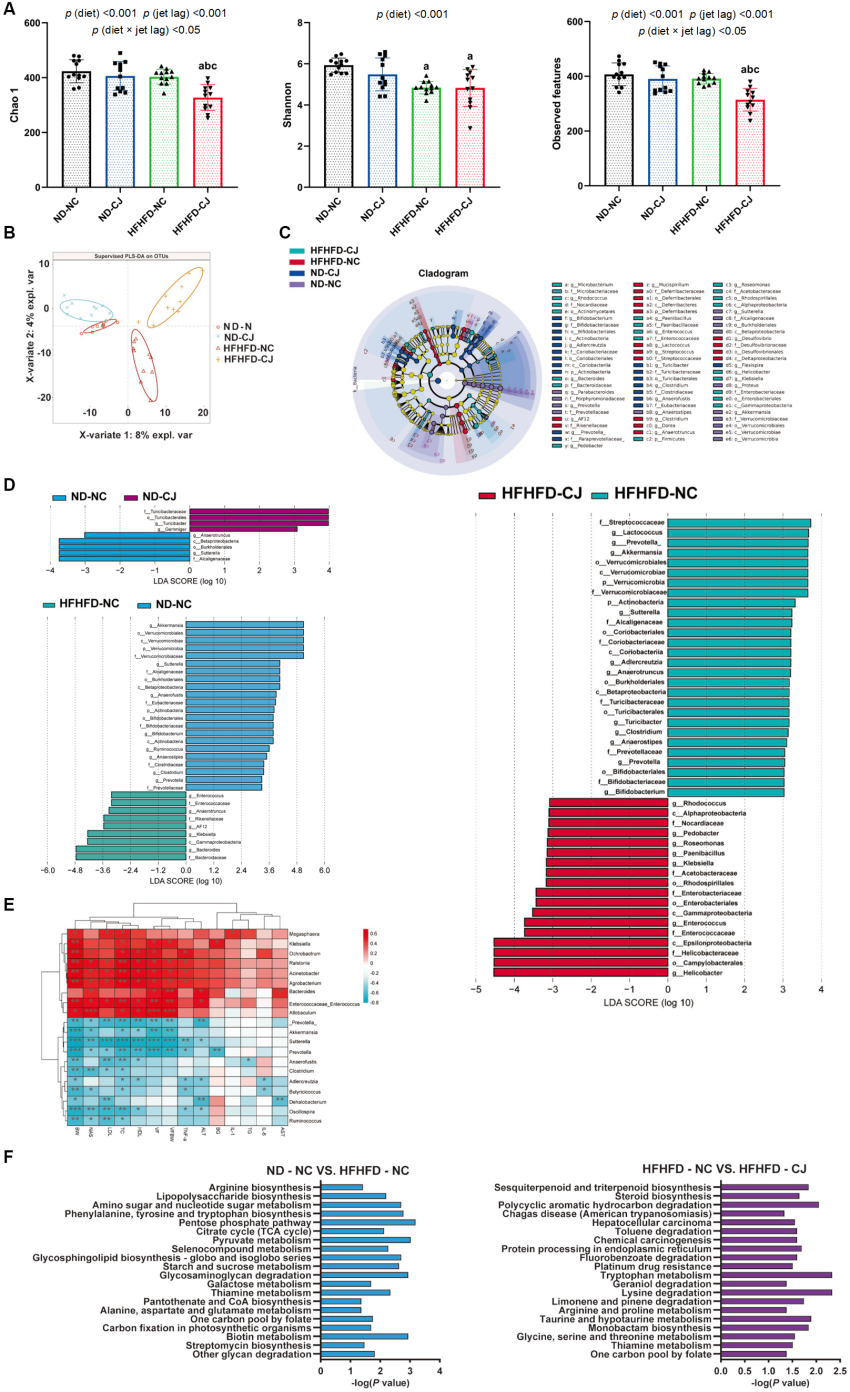
Chronic jet lag altered the composition of gut microbiota in mice. **(A)** Alpha diversity-related index: Chao1, Shannon, and Observed features; **(B)** PLS-DA: Partial Least Squares Discriminant Analysis; **(C,D)** Cladogram of LEfSe analysis and scores of taxonomic biomarkers identified by linear discriminant analysis (LDA) using LEfSe in ND-fed mice, NC mice and HFHFD-fed mice, LDA value >3 were showed in the figures; **(E)** the correlation heatmap of the relative abundance of bacteria at genus level and indicators of metabolic abnormalities; **(F)** KEGG pathway analysis in NC mice and HFHFD-fed mice. Differences were determined using a two-way ANOVA followed by a Tukey’s multiple comparisons test **(A)**. Data were expressed as Mean ± SD, *n* = 12 per group, *^a^p* < 0.05, vs. ND-NC group; *^b^p* < 0.05, vs. ND-CJ group; *^c^p* < 0.05, vs. HFHFD-NC group, ^*^p < 0.05, ^**^p < 0.01, ^***^p < 0.001. *p* (diet), main effect of diet; *p* (jet leg), main effect of jet leg; *p* (light × jet leg), interaction effect of jet leg and diet. BW, body weight; VF, visceral fat; BG, blood glucose; NAS, NAFLD activity score; TG, triglyceride; TC, total cholesterol; LDL, low-density lipoprotein; HDL, high-density lipoprotein; AST, aspartate aminotransferase; ALT, alanine aminotransferase.

The Chao1 and observed features of the gut microbiome were decreased in the HFHFD-CJ group vs. the HFHFD-NC group (Figure 3A). Main effects of diet (*p* < 0.001) and jet lag (*p* < 0.001), as well as diet × jet lag interactive effect (*p* < 0.05) were observed for Chao 1 and observed features. PLS-DA analysis revealed that there were significant differences among groups, indicating that CJ caused significant alterations in the β-diversity of the gut microbiome in mice (Figure 3B). LEfSe analysis showed higher abundances of *Turicibacter* and *Gemmiger* genera and lower abundances of *Anaerotruncus* and *Sutterella* genera in the ND-CJ group vs. ND-NC group. Compared with the ND-NC group, increased abundance of *Enterococcus, Anaerotruncus, Klebsiella,* and *Bacteroides* genera and decreased abundance of *Akkermansia*, *Sutterella, Anaerofustis*, *Bifidobacterium, Ruminococcus, Clostridium*, and *Prevotella* genera were observed in the HFHFD-NC group. Compared with the HFHFD-NC group, the HFHFD-CJ group had relatively lower abundances of *Akkermansia*, *Lactococcus*, *Prevotella*, *Clostridium*, and *Bifidobacterium* genera (Figures 3C,D).

The genera *Enterococcus* and *Allobaculum* were positively correlated with body weight, serum lipids, and NAS, whereas the genera *Prevotella, Akkermansia*, *Clostridium, Oscillospira*, and *Ruminococcus* negatively correlated with body weight, serum lipids, and NAS (Figure 3E). The genus *Bacteroides* was positively correlated with serum ALT and AST, whereas *Dehalobacterium* was inversely correlated with serum ALT and AST levels (Figure 3E).

KEGG analysis showed that HFHFD caused alterations in metabolic pathways of gut microbiota, such as biotin, amino acid, and carbohydrate metabolism (Figure 3F), and CJ upregulated pathways related to chemical carcinogenesis, hepatocellular carcinoma, and steroid biosynthesis in HFHFD-fed mice (Figure 3F).

### 3.5 Changes in rhythm of colon microbiota in mice

**Figure 4:**
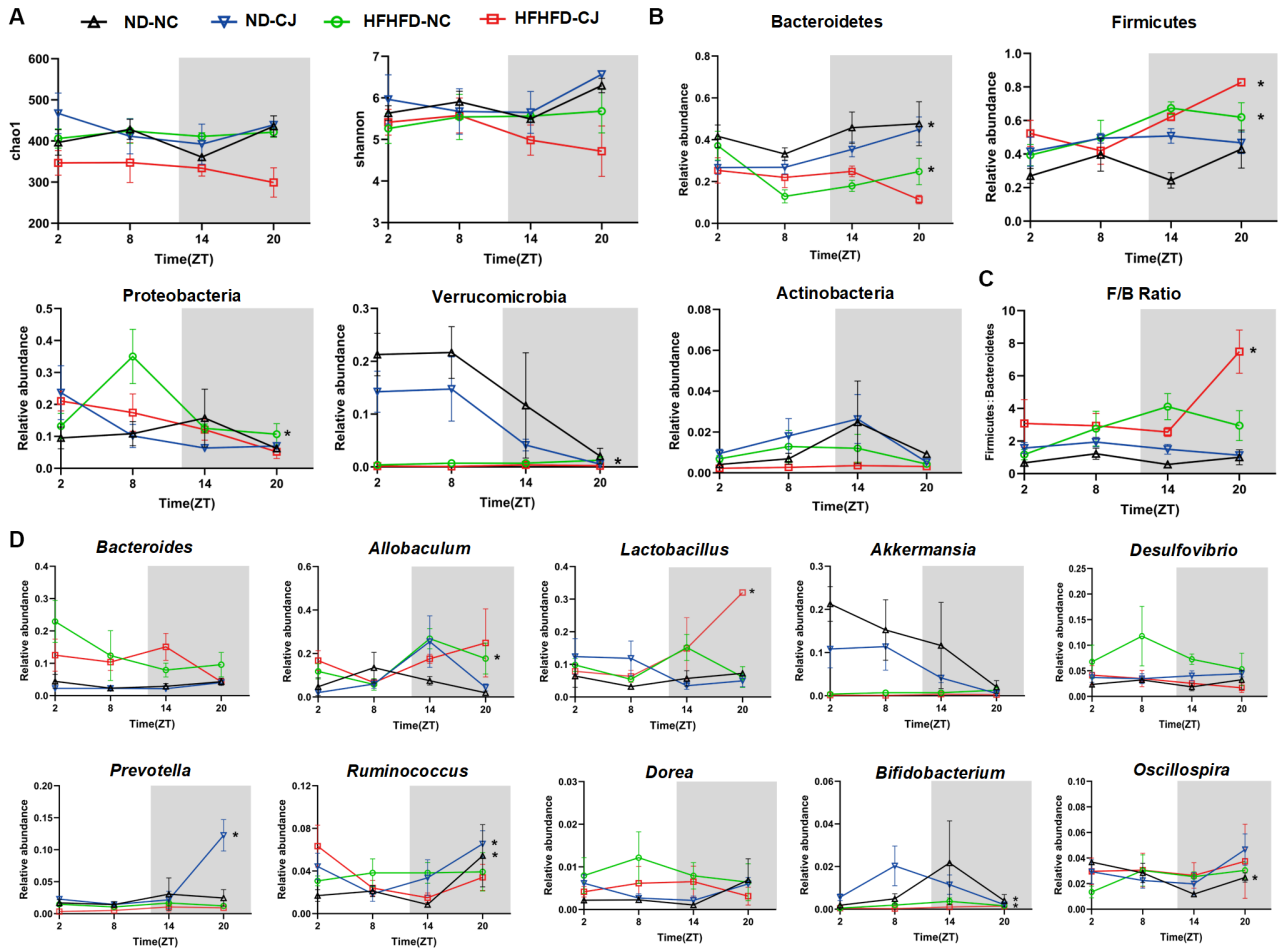
Chronic jet lag altered the rhythm of gut microbiota in mice. The rhythm of **(A)** α diversity, **(B)** the phylum level, **(C)** ratio of Firmicutes to Bacteroidetes, and **(D)** the genus level in bacteria. Data are represented as mean ± SEM, n = 3 per group at each time point. Asterisks indicate the phylum or genera that exhibit significant oscillatory rhythmicity using the *Cosinor* analysis with a *value of p* of less than 0.05.

No diurnal variations were observed in the alpha diversity of the colon microbiota (Figure 4A). Bacteroidetes, Firmicutes, Proteobacteria, Verrucomicrobia, and Actinobacteria were the main phyla present in each group. Among them, they did not show rhythmicity in the ND-NC group, whereas CJ caused rhythmic oscillation of Bacteroidetes and Verrucomicrobia, with Bacteroidetes peaking at night and Verrucomicrobia reaching a trough at night. HFHFD feeding caused significant oscillation patterns in Bacteroidetes, Firmicutes, and Proteobacteria, with Bacteroidetes peaking in the morning, Firmicutes reaching a trough in the morning, and Proteobacteria peaking in the afternoon (Figure 4B). In addition, Firmicutes and the ratio of Firmicutes to Bacteroidetes (F/B ratio) acquired rhythmicity in the HFHFD-CJ group and both peaked at night (Figure 4C).

At the genus level, *Oscillospira* exhibited rhythmicity in the ND-NC group, peaking in the morning. In the ND-CJ group, *Prevotella, Ruminococcus,* and *Bifidobacterium* showed diurnal rhythmicity, with *Prevotella* and *Ruminococcus* peaking at night and *Bifidobacterium* reaching a trough at night, whereas *Allobaculum* and *Dorea* diurnal variation approached significance (*p* = 0.057, *p* = 0.057). In the HFHFD-NC group, *Allobaculum* and *Bifidobacterium* showed diurnal oscillations and both peaked in the evening. In the HFHFD-CJ group, *Lactobacillus* exhibited diurnal patterns, peaking at night. Remarkably, *Bacteroides, Akkermansia*, and *Desulfovibrio* displayed no significant daily rhythms in any of the groups (Figure 4D).

### 3.6 Changes in the composition of colon mycobiome in mice

**Figure 5:**
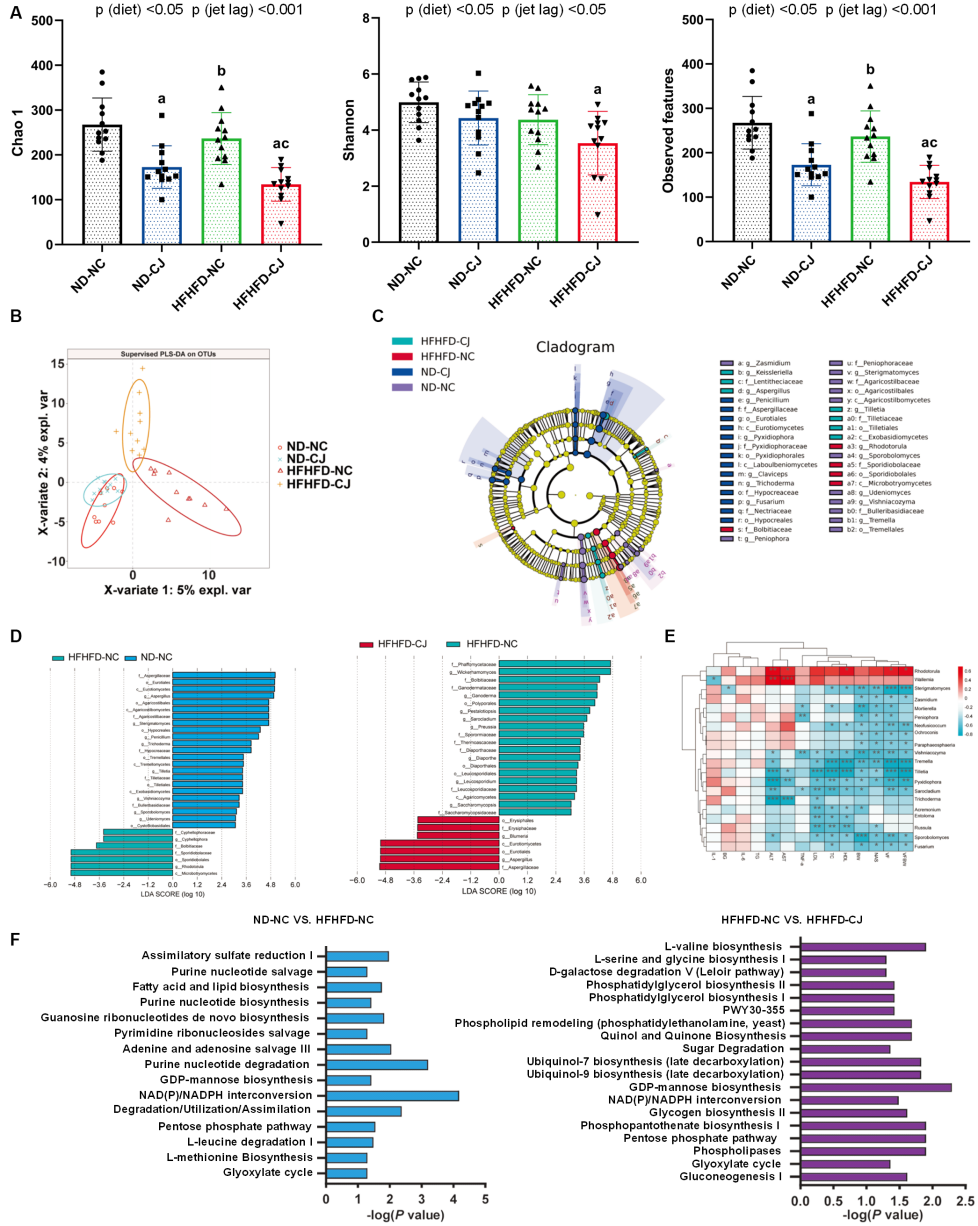
Chronic jet lag altered the composition of gut mycobiome in mice. **(A)** Alpha diversity-related index: Chao1, Shannon, and Observed features; **(B)** PLS-DA: Partial Least Squares Discriminant Analysis; **(C,D)** Cladogram of LEfSe analysis and scores of taxonomic biomarkers identified by linear discriminant analysis (LDA) using LEfSe in NC mice and HFHFD-fed mice, LDA value >3 were showed in the figure; **(E)** the correlation heatmap of the relative abundance of fungi at genus level and indicators of metabolic abnormalities; **(F)** MetaCyc pathway analysis in NC mice and HFHFD-fed mice. Differences were determined using a two-way ANOVA followed by a Tukey’s multiple comparisons test **(A)**. Data were expressed as Mean ± SD, *n* = 12 per group, ^a^*p* < 0.05, vs. ND-NC group; ^b^*p* < 0.05, vs. ND-CJ group; ^c^*p* < 0.05, vs. HFHFD-NC group. *p < 0.05, **p < 0.01, ***p < 0.001. *p* (diet), main effect of diet; *p* (jet leg), main effect of jet leg; *p* (light × jet leg), interaction effect of jet leg and diet. BW, body weight; VF, visceral fat; BG, blood glucose; NAS, NAFLD activity score; TG, triglyceride; TC, total cholesterol; LDL, low-density lipoprotein; HDL, high-density lipoprotein; AST, aspartate aminotransferase; ALT, alanine aminotransferase.

Compared with the HFHFD-NC and ND-NC groups, the Chao1 and observed features of gut fungi were significantly decreased in the HFHFD-CJ group, with significant main effects of diet (*p* < 0.05) and jet lag (*p* < 0.001) noted (Figure 5A). There was a structural difference among the groups according to the PLS-DA analysis (Figure 5B). According to LEfSe analysis, no differential genera were found between the ND-NC and ND-CJ groups, while the HFHFD-NC group had increased abundances of *Rhodotorula* and *Cyphellophora* genera, and decreased abundances of *Aspergillus*, *Sterigmatomyces*, *Penicillium, Trichoderme, Tilletia, Vishniacozyma, Sporobolomyces* genera vs. ND-NC group. In HFHFD-fed mice, there was an increase in the genera *Aspergillus* and *Blumeria*, and a decrease in the genera *Wickerhamomyces, Ganoderma,* and *Saccharomycopsis* in the HFHFD-CJ vs. HFHFD-NC groups (Figures 5C,D). The genera *Rhodotorula* and *Wallemia* positively correlated with serum AST and ALT, whereas the genera *Tilletia, Pyxidiophora,* and *Trichoderme* were negatively correlated with NAS, AST, and ALT. The genera *Sterigmatomyces, Mortierella, Sporobolomyces, Vishniacozyma,* and *Fusarium* negatively correlated with body weight, TC, and NAS (Figure 5E).

Pathway analysis of the MetaCyc database showed that pathways related to fatty acid and lipid biosynthesis, nucleotide metabolism, glyoxylate cycle, pentose phosphate pathway (PPP), and NAD (P) / NADPH conversion in gut fungi were altered in the HFHFD-NC group vs. ND-NC group (Figure 5F). CJ further altered the pathways related to the glyoxylate cycle, PPP, and NAD (P) / NADPH conversion in the HFHFD mice (Figure 5F).

### 3.7 Changes in rhythm of colon mycobiome in mice

**Figure 6:**
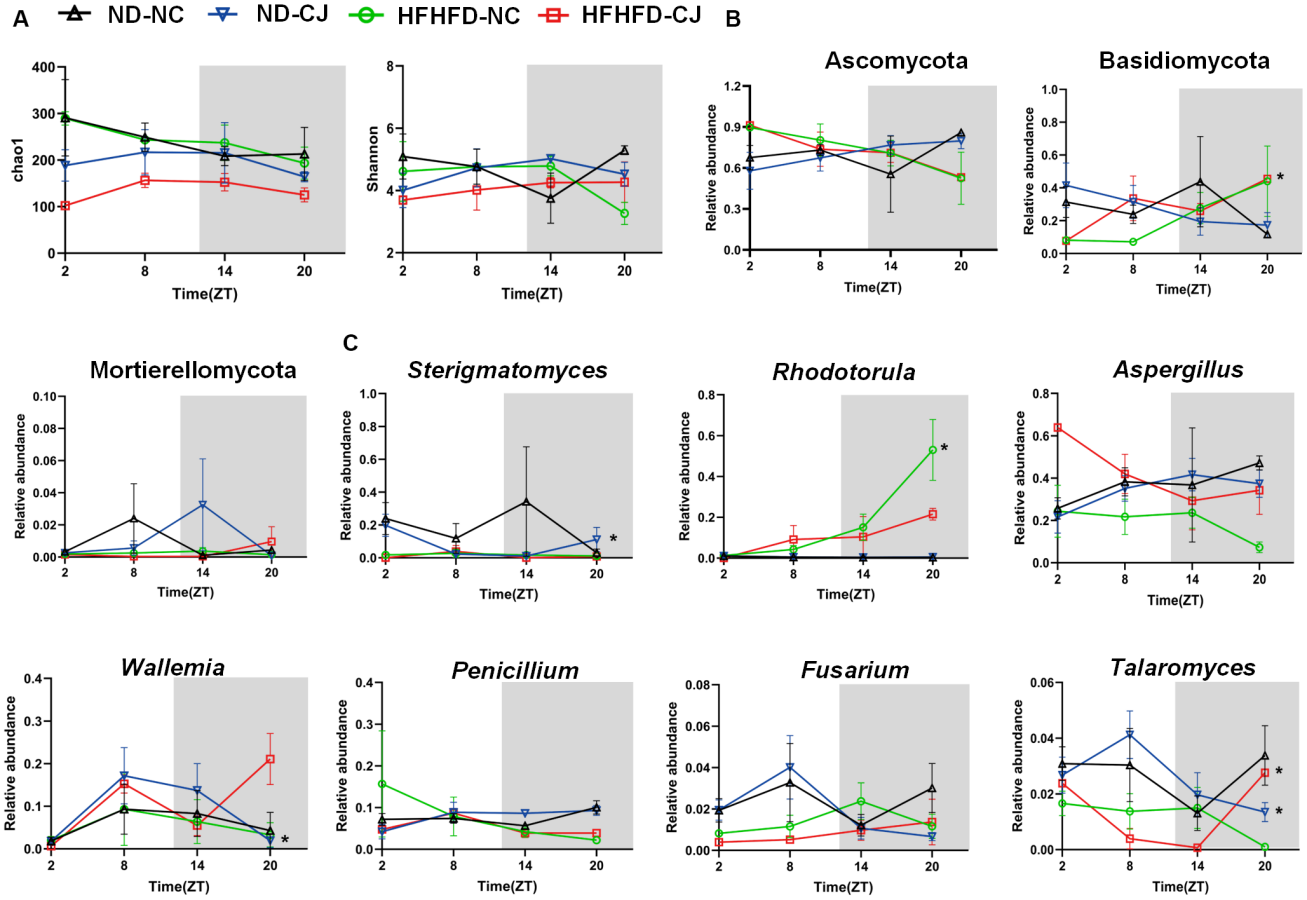
Chronic jet lag altered the rhythm of gut mycobiome in mice. The rhythm of **(A)** α diversity, **(B)** the phylum level, **(C)** the genus level in fungi. Data are represented as mean ± SEM, n = 3 per group at each time point. Asterisks indicate the phylum or genera that exhibit significant oscillatory rhythmicity using the *Cosinor* analysis with a *value of p* of less than 0.05.

There were no rhythmic changes in the alpha diversity of gut fungi (Figure 6A). Ascomycota, Basidiomycota, and Mortierellomycota were the main phyla present in each group. Among them, Basidiomycota showed a diurnal pattern in the HFHFD-NC group, peaking at night (Figure 6B). At the genus level, we focused on highly abundant fungi that were present in all samples (Figure 6C). None of them in the ND-NC group exhibited significant oscillation patterns. In the ND-CJ group, the abundance of *Sterigmatomyces*, *Wallemia*, and *Talaromyces* demonstrated significant fluctuations, with *Sterigmatomyces* peaking in the morning and *Wallemia* and *Talaromyces* peaking in the afternoon. In the HFHFD-NC group, *Rhodotorula* exhibited daily rhythmicity and peaked at night. In the HFHFD-CJ group, *Talaromyces* exhibited rhythmicity and peaked at night, and *Penicillium* rhythmicity approached statistical significance (*p* = 0.055).

### 3.8 Interactions between the gut fungal and bacterial communities

**Figure 7:**
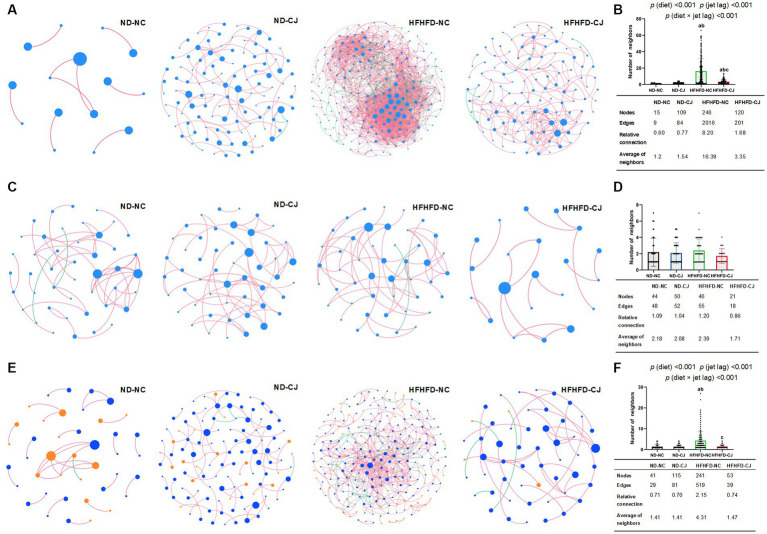
Correlation networks of gut microbiota. Correlation networks among microbiota were built using the Spearman correlation test in the four study groups. **(A)** Abundance correlation networks among gut bacteria are shown. Each node represents an ASV sequence, and its size is scaled to the number of edges within each network. Edges indicate correlations (positive in red and negative in green). Only ASV sequences present in more than 50% of samples in the group were considered, and only significant correlations (*p* < 0.05) are shown. The table in the inset shows the network parameters. The relative connectedness is the ratio between the number of edges and the number of nodes in the network. **(B)** Neighbors of each node within the network among bacteria. **(C)** Abundance correlation networks among gut fungi are shown. **(D)** Neighbors of each node within the network among fungi. **(E)** Abundance correlation networks between gut bacteria and fungi are shown. Each node represents an ASV sequence, with bacteria in blue and fungi in orange. **(F)** Neighbors of each node within the network between bacteria and fungi. Differences were determined using a two-way ANOVA followed by a Tukey’s multiple comparisons test. Data were expressed as Mean ± SD, ^a^*p* < 0.05, vs. ND-NC group; ^b^*p* < 0.05, vs. ND-CJ group; ^c^*p* < 0.05, vs. HFHFD-NC group. *p* (diet), main effect of diet; *p* (jet leg), main effect of jet leg; *p* (light × jet leg), interaction effect of jet leg and diet.

Microbial abundance correlation networks were constructed to evaluate ecosystem structure. The bacterial networks differed among the groups (Figures 7A,B). A simple network of correlations between the bacteria was observed in the ND-NC group. In comparison, the density of the bacterial correlation network increased in the HFHFD-NC group, as indicated by an increased relative connectedness and a higher number of neighbors. There was no statistically significant difference between the ND-NC and ND-CJ groups for these network density-related parameters, but the network in the ND-CJ group had more nodes (ASV sequences) and edges (connections). Compared to the HFHFD-NC group, the density of the bacterial network was lower in the HFHFD-CJ group. Main effects of diet (*p* < 0.001) and jet lag (*p* < 0.001), as well as interactive effect between diet and jet lag (*p* < 0.001) were observed for number of neighbors in bacterial network. There was no significant difference in the network density among the groups in the fungal interaction network (Figures 7C,D).

Abundance correlation networks of bacterial and fungal interactions were constructed to explore the interkingdom interactions (Figures 7E,F). Compared with the ND-NC group, the other three groups had a more complex fungi-bacterial network, as illustrated by increased nodes and edges. Moreover, more neighbors were observed for each node in the HFHFD-NC group than in the other three groups [*p* (diet) <0.001, *p* (jet lag) <0.001].

## 4 Discussion

Proper circadian rhythm is an important feature of normal health, enabling organisms to adapt to daily environmental changes. Jet lag, which leads to repeated phase shifts of circadian clock, is experienced by more than transmeridian travellers. It is also experienced by shift workers, individuals with evening preferences, and those who sleep short on workdays, then stay up later but sleep longer on weekends (which is also coined as social jet lag) worldwide (Roenneberg and Merrow, 2016). In the present animal experiment, the results suggest that CJ, induced by advancing the light–dark cycle over long periods, promotes obesity and MAFLD progression in an HFHFD mouse model, which is consistent with our previous observations that circadian disruption is a novel risk factor for MAFLD progression (Wei et al., 2020). More importantly, this study demonstrated that CJ altered the abundance, composition, and rhythm of the gut microbiome and mycobiome and their interactions. To the best of our knowledge, this is the first study to explore the effects of circadian disruption on gut mycobiome.

Changes in the gut bacterial microbiome in MAFLD have been extensively studied. In the HFHFD-NC group, we found increases in *Enterococcus, Anaerotruncus,* and *Bacteroides* genera and decreases in *Akkermansia*, *Bifidobacterium, Ruminococcus, Clostridium*, and *Prevotella* genera in comparison to the ND-NC group, which agrees with previous studies (Pataky et al., 2016; Juarez-Fernandez et al., 2021). Notably, CJ resulted in even lower abundances of *Akkermansia*, *Prevotella*, *Clostridium*, and *Bifidobacterium* in the HFHFD-CJ group vs. the HFHFD-NC group. *Akkermansia*, *Prevotella*, and *Clostridium* have been associated with improved gut barrier function, and studies have demonstrated their protective effects against metabolic disorders (Everard et al., 2013; Leung et al., 2016; Pan et al., 2019). In MAFLD patients, negative correlations between the severity of MAFLD and the abundance of *Akkermansia*, *Prevotella*, and *Clostridium* have been reported (Pataky et al., 2016; Han et al., 2022). Our animal experiment also demonstrated negative correlations between NAS and the abundance of the genera *Akkermansia*, *Prevotella,* and *Clostridium*.

Studies investigating the gut mycobiome in mouse models are scarce. In the present study, the HFHFD-NC group had increased abundances of *Rhodotorula* and *Cyphellophora* genera, and decreased abundances of *Aspergillus*, *Sterigmatomyces*, *Penicillium* vs. ND-NC groups, and CJ resulted in increased abundances of *Aspergillus*, *Blumeria* and decreased abundances of *Wickerhamomyces, Ganoderma,* and *Saccharomycopsis* in HFHFD-fed mice. Correlation analysis showed that *Rhodotorula* and *Wallemia* were positively correlated with AST and ALT, indicating that these genera may be related to liver damage. Among them, *Rhodotorula* spp. not only has the capability to synthesize saturated long-chain fatty acids such as palmitic acid (Hof, 2017), but also exhibits the ability to synthesize lipids through the utilization of glucose, acetic acid, and propionic acid (Xue et al., 2018). Additionally, Rodriguez et al. related *Rhodotorula* to metabolic abnormalities linked to lipid alterations (Mar Rodriguez et al., 2015). In line with this, Ricardo García-Gamboa et al. identified a positive correlation between *Rhodotorula* spp. and weight, BMI, and fat mass (García-Gamboa et al., 2021). These findings may indicate a potential association between *Rhodotorula* and MAFLD. *Wallemia* has been considered the primary causative agent of asthma and other allergological problem(Skalski et al., 2018), and MAFLD and obesity influence the incidence rates of asthma(Michalovich et al., 2019; Rastogi, 2020). Pathway analysis of the MetaCyc database in gut fungi revealed that the pentose phosphate pathway (PPP) was upregulated in HFHFD-NC mice and further upregulated in HFHFD-CJ mice. The involvement of the PPP in immunometabolic regulation and fatty acid synthesis has been established (Muri and Kopf, 2020), but whether PPP up-regulation in gut fungi is associated with host immune disorders and metabolic disorders is not clear, and more research is needed in the future.

It has been reported that the gut bacterial composition oscillates in a diurnal pattern (Thaiss et al., 2014), although the exact oscillation regularities of specific phyla are not well studied, and the rhythmic changes in gut microbiota are not consistent among studies. For example, for Bacteroidetes and Firmicutes, some found rhythmicity (Zarrinpar et al., 2014), while others did not (Ye et al., 2020). It should be emphasized that in both human and animal models, the rhythmic OTUs only accounted for a small proportion, generally no more than 20% (Zarrinpar et al., 2014; Reitmeier et al., 2020). Thaiss et al. proposed that non-oscillating species, which are not subject to circadian rhythms and exist in relatively stable abundance in the gut, would represent a population responsible for “housekeeping” functions (Thaiss et al., 2014). In the present study, ND-NC mice did not show rhythmicity in the main phyla, while HFHFD and/or CJ caused cyclical fluctuations, indicating that the stable state exhibited in ND-NC mice was disturbed by HFHFD and/or CJ.

Few studies have examined the rhythms of the gut fungi. Here, we found that gut fungi, like bacteria, showed no circadian rhythm in the ND-NC group, but the abundance of some gut fungi fluctuated in the ND-CJ and HFHFD-fed groups. Fungal cells are 100 times larger in volume than typical bacterial cells, and they provide abundant biomaterials and unique metabolic functions to the microbial community. Moreover, fungi provide the surface area for host–microbe interactions (Huffnagle and Noverr, 2013). Therefore, we speculate that gut fungi in a relatively stable state are beneficial for maintaining the overall gut microbial community homeostasis, while fluctuations of gut fungi may be detrimental to the host. Indeed, modulation of the gut fungal community structure has been associated with gut inflammation, which plays a pivotal role in the pathogenesis of MAFLD (Chehoud et al., 2015). Thus, fungal fluctuations may contribute to the development of MAFLD in the ND-CJ group and HFHFD-fed groups.

Fungi and bacteria coexist and interact with one another. Gut fungi affect bacterial colonization (Kombrink et al., 2019; Fernandez de Ullivarri et al., 2020). In turn, bacteria regulate fungal germination and hyphal growth (Noverr and Huffnagle, 2004; Garcia et al., 2017). Here, we found the existence of a wide range of bacterial and fungal interactions in the gut, both inter- and intra-kingdoms. Interestingly, under the intervention of HFHFD and/or CJ, we found that the fungi-bacteria interaction network became more complex. It has been proposed that microbial communities with lower complexities and weaker interactions are more likely to remain stable (Yonatan et al., 2022). Our data suggest that under the intervention of an HFHFD and/or CJ, gut microbial stability is disrupted and may be more vulnerable to external environmental influences. However, the significance of gut fungi-bacteria interactions is not yet fully understood, and their relationship with the development of MAFLD requires further study.

Although important discoveries were made in our study, there are several limitations. First, it was an observational and descriptive study with a relatively small sample size. Second, we experimented only with male mice. Sex is thought to be an important factor in MAFLD and its outcomes (Zhang et al., 2021). Moreover, it has been reported that there are sex differences in response to circadian disruption recently (Anderson et al., 2023). Future studies are needed to focus on the effects of CJ on gut bacterial and fungal rhythms in female subjects. Finally, there was no comparison between the gut microbiota of mice undergoing CJ and those in recovery. It remains an open question whether the gut microbiota will recover and how long it will take; thus, more experiments are needed to assess the effects of chronic circadian rhythm disruption on gut microbial abundance and rhythm, as well as network organization.

## 5 Conclusion

Our results revealed that CJ altered the composition and structure of the gut bacteria and fungi, disrupted the rhythmic oscillation of the gut microbiota and mycobiome, affected interactions among the gut microbiome, and promoted the progression of MAFLD in HFHFD mice. Circadian disruptions have become increasingly prevalent in modern society. Our study highlights that circadian disruption is a novel risk factor for MAFLD progression. Circadian disruption or jet lag is sometimes inevitable. With the better understanding of the alterations and roles of gut microbiome and mycobiome in MAFLD, microbiome-targeted interventions (e.g., probiotics, prebiotics, synbiotics, fecal microbiota transplantation, engineered bacteria, *et al*) and fungi-based therapeutical strategies (e.g., antifungal drugs, fungal prebiotics, fungal products, *et al*) have been proposed as new approaches for the treatment of MAFLD (Aron-Wisnewsky et al., 2020b; Xue et al., 2022; Szóstak et al., 2023). The work presented here further underscores that modulating the gut microbiota and mycobiome represents a novel and promising strategy for treating diseases related to circadian disruption. Additionally, our study highlights the need to consider the role of gut fungi, timing of sample collection, and timing of intervention for microbiome-targeted therapies for metabolic diseases in scientific research and clinical practice.

## Data availability statement

The datasets presented in this study can be found in online repositories. The names of the repository/repositories and accession number(s) can be found at: https://www.ncbi.nlm.nih.gov/, PRJNA968141; PRJNA1018124.

## Ethics statemen

The animal study was approved by Animal Use and Care Committee of Central South University. The study was conducted in accordance with the local legislation and institutional requirements.

## Author contributions

RZ: Software, Visualization, Writing – original draft, Writing – review & editing. XX: Conceptualization, Methodology, Visualization, Writing – original draft, Writing – review & editing. YS: Data curation, Validation, Visualization, Writing – original draft. AQ: Conceptualization, Methodology, Validation, Writing – review & editing. XL: Data curation, Methodology, Software, Writing – review & editing. JX: Methodology, Software, Writing – review & editing. RR: Data curation, Visualization, Writing – review & editing. DZ: Conceptualization, Funding acquisition, Methodology, Resources, Writing – original draft, Writing – review & editing.

## Glossary

ALT: alanine aminotransferase
AST: aspartate aminotransferase
ASV: amplicon sequence variant
AUC: area under the curve
BG: blood glucose
BW: body weight
CJ: chronic jet lag
FBG: fasting blood glucose
HDL-C: high-density lipoprotein cholesterol
HFHFD: high-fat and high-fructose diet
IL-1β: Interleukin-1β
IL-6: Interleukin-6
IPGTT: intraperitoneal glucose tolerance test
ITS: internal transcribed spacer
LDL-C: low-density lipoprotein cholesterol
MAFLD: metabolic dysfunction associated fatty liver disease
NAFLD: nonalcoholic fatty liver disease
NAS: NAFLD activity scores
NC: normal circadian rhythm
ND: normal diet
OTU: operational taxonomic unit
PLS-DA: partial least squares discriminant analysis
qRT-PCR: quantitative real-time polymerase chain reaction
TC: total cholesterol
TG: Triglycerides
TNF-α: tumor necrosis factor-α
ZT: zeitgeber time

## References

[ref1] Anderson S. T. Meng H. Brooks T. G. Tang S. Y. Lordan R. Sengupta A.. (2023). Sexual dimorphism in the response to chronic circadian misalignment on a high-fat diet. Sci. Transl. Med. 15:eabo2022. doi: 10.1126/scitranslmed.abo2022, PMID: 37196066

[ref2] Aron-Wisnewsky J. Vigliotti C. Witjes J. Le P. Holleboom A. G. Verheij J.. (2020a). Gut microbiota and human NAFLD: disentangling microbial signatures from metabolic disorders. Nat. Rev. Gastroenterol. Hepatol. 17, 279–297. doi: 10.1038/s41575-020-0269-9, PMID: 32152478

[ref3] Aron-Wisnewsky J. Warmbrunn M. V. Nieuwdorp M. Clément K. (2020b). Nonalcoholic fatty liver disease: modulating gut microbiota to improve severity? Gastroenterology 158, 1881–1898. doi: 10.1053/j.gastro.2020.01.049, PMID: 32044317

[ref4] Bishehsari F. Voigt R. M. Keshavarzian A. (2020). Circadian rhythms and the gut microbiota: from the metabolic syndrome to cancer. Nat. Rev. Endocrinol. 16, 731–739. doi: 10.1038/s41574-020-00427-4, PMID: 33106657 PMC8085809

[ref5] Buijs F. N. Leon-Mercado L. Guzman-Ruiz M. Guerrero-Vargas N. N. Romo-Nava F. Buijs R. M. (2016). The circadian system: a regulatory feedback network of periphery and brain. Physiology (Bethesda) 31, 170–181. doi: 10.1152/physiol.00037.2015, PMID: 27053731

[ref6] Chehoud C. Albenberg L. G. Judge C. Hoffmann C. Grunberg S. Bittinger K.. (2015). Fungal signature in the gut microbiota of Pediatric patients with inflammatory bowel disease. Inflamm. Bowel Dis. 21, 1948–1956. doi: 10.1097/MIB.0000000000000454, PMID: 26083617 PMC4509842

[ref7] Demir M. Lang S. Hartmann P. Duan Y. Martin A. Miyamoto Y.. (2022). The fecal mycobiome in non-alcoholic fatty liver disease. J. Hepatol. 76, 788–799. doi: 10.1016/j.jhep.2021.11.029, PMID: 34896404 PMC8981795

[ref8] Doron I. Leonardi I. Li X. V. Fiers W. D. Semon A. Bialt-DeCelie M.. (2021). Human gut mycobiota tune immunity via CARD9-dependent induction of anti-fungal IgG antibodies. Cells 184, 1017–1031.e14. doi: 10.1016/j.cell.2021.01.016, PMID: 33548172 PMC7936855

[ref9] Eslam M. Newsome P. N. Sarin S. K. Anstee Q. M. Targher G. Romero-Gomez M.. (2020). A new definition for metabolic dysfunction-associated fatty liver disease: an international expert consensus statement. J. Hepatol. 73, 202–209. doi: 10.1016/j.jhep.2020.03.039, PMID: 32278004

[ref10] Everard A. Belzer C. Geurts L. Ouwerkerk J. P. Druart C. Bindels L. B.. (2013). Cross-talk between Akkermansia muciniphila and intestinal epithelium controls diet-induced obesity. Proc. Natl. Acad. Sci. U. S. A. 110, 9066–9071. doi: 10.1073/pnas.1219451110, PMID: 23671105 PMC3670398

[ref11] Faraut B. Cordina-Duverger E. Aristizabal G. Drogou C. Gauriau C. Sauvet F.. (2022). Immune disruptions and night shift work in hospital healthcare professionals: the intricate effects of social jet-lag and sleep debt. Front. Immunol. 13:939829. doi: 10.3389/fimmu.2022.939829, PMID: 36164341 PMC9509137

[ref12] Fernandez de Ullivarri M. Arbulu S. Garcia-Gutierrez E. Cotter P. D. (2020). Antifungal peptides as therapeutic agents. Front. Cell. Infect. Microbiol. 10:105. doi: 10.3389/fcimb.2020.00105, PMID: 32257965 PMC7089922

[ref13] Fotis D. Liu J. Dalamaga M. (2022). Could gut mycobiome play a role in NAFLD pathogenesis? Insights and therapeutic perspectives. Metabolism Open 14:100178. doi: 10.1016/j.metop.2022.100178, PMID: 35308892 PMC8927988

[ref14] Garcia C. Tebbji F. Daigneault M. Liu N. N. Kohler J. R. Allen-Vercoe E.. (2017). The human gut microbial metabolome modulates fungal growth via the TOR Signaling pathway. mSphere 2:e00555-17. doi: 10.1128/mSphere.00555-17 29242837 PMC5729221

[ref15] García-Gamboa R. Kirchmayr M. R. Gradilla-Hernández M. S. Pérez-Brocal V. Moya A. González-Avila M. (2021). The intestinal mycobiota and its relationship with overweight, obesity and nutritional aspects. J. Hum. Nutr. Diet. 34, 645–655. doi: 10.1111/jhn.12864, PMID: 33586805

[ref16] Han Y. Li L. Wang B. (2022). Role of *Akkermansia muciniphila* in the development of nonalcoholic fatty liver disease: current knowledge and perspectives. Front. Med. 16, 667–685. doi: 10.1007/s11684-022-0960-z, PMID: 36318353

[ref17] Hof H. (2017). Fungi in the gut - the gut mycobiome. Z. Gastroenterol. 55, 772–778. doi: 10.1055/s-0043-112657 28799153

[ref18] Huffnagle G. B. Noverr M. C. (2013). The emerging world of the fungal microbiome. Trends Microbiol. 21, 334–341. doi: 10.1016/j.tim.2013.04.002, PMID: 23685069 PMC3708484

[ref19] Juarez-Fernandez M. Porras D. Petrov P. Roman-Saguillo S. Garcia-Mediavilla M. V. Soluyanova P.. (2021). The Synbiotic combination of Akkermansia muciniphila and quercetin ameliorates early obesity and NAFLD through gut microbiota reshaping and bile acid metabolism modulation. Antioxidants (Basel) 10:2001. doi: 10.3390/antiox10122001, PMID: 34943104 PMC8698339

[ref20] Kapitan M. Niemiec M. J. Steimle A. Frick J. S. Jacobsen I. D. (2019). Fungi as part of the microbiota and interactions with intestinal bacteria. Curr. Top. Microbiol. Immunol. 422, 265–301. doi: 10.1007/82_2018_117 30062595

[ref21] Kleiner D. E. Brunt E. M. Van Natta M. Behling C. Contos M. J. Cummings O. W.. (2005). Design and validation of a histological scoring system for nonalcoholic fatty liver disease. Hepatology 41, 1313–1321. doi: 10.1002/hep.20701, PMID: 15915461

[ref22] Kolde R. (2019). Pheatmap: pretty Heatmaps. R package version 1.0.12. Available at: https://cran.r-project.org/package=pheatmap

[ref23] Kombrink A. Tayyrov A. Essig A. Stockli M. Micheller S. Hintze J.. (2019). Induction of antibacterial proteins and peptides in the coprophilous mushroom Coprinopsis cinerea in response to bacteria. ISME J. 13, 588–602. doi: 10.1038/s41396-018-0293-8, PMID: 30301946 PMC6461984

[ref24] Le Roy T. Llopis M. Lepage P. Bruneau A. Rabot S. Bevilacqua C.. (2013). Intestinal microbiota determines development of non-alcoholic fatty liver disease in mice. Gut 62, 1787–1794. doi: 10.1136/gutjnl-2012-303816 23197411

[ref25] Leung C. Rivera L. Furness J. B. Angus P. W. (2016). The role of the gut microbiota in NAFLD. Nat. Rev. Gastroenterol. Hepatol. 13, 412–425. doi: 10.1038/nrgastro.2016.85 27273168

[ref27] Maas E. Penders J. Venema K. (2023). Fungal-bacterial interactions in the human gut of healthy individuals. J. Fungi 9:139. doi: 10.3390/jof9020139, PMID: 36836254 PMC9965947

[ref28] Mar Rodriguez M. Perez D. Javier Chaves F. Esteve E. Marin-Garcia P. Xifra G.. (2015). Obesity changes the human gut mycobiome. Sci. Rep. 5:14600. doi: 10.1038/srep14600, PMID: 26455903 PMC4600977

[ref29] Mbaye B. Borentain P. Magdy Wasfy R. Alou M. T. Armstrong N. Mottola G.. (2022). Endogenous ethanol and triglyceride production by gut Pichia kudriavzevii, Candida albicans and Candida glabrata yeasts in non-alcoholic steatohepatitis. Cells 11:3390. doi: 10.3390/cells11213390, PMID: 36359786 PMC9654979

[ref30] Michalovich D. Rodriguez-Perez N. Smolinska S. Pirozynski M. Mayhew D. Uddin S.. (2019). Obesity and disease severity magnify disturbed microbiome-immune interactions in asthma patients. Nat. Commun. 10:5711. doi: 10.1038/s41467-019-13751-9, PMID: 31836714 PMC6911092

[ref31] Mukherji A. Dachraoui M. Baumert T. F. (2020). Perturbation of the circadian clock and pathogenesis of NAFLD. Metabolism 111:154337. doi: 10.1016/j.metabol.2020.154337, PMID: 32795560 PMC7613429

[ref32] Murakami M. Tognini P. (2020). The circadian clock as an essential molecular link between host physiology and microorganisms. Front. Cell. Infect. Microbiol. 9:469. doi: 10.3389/fcimb.2019.00469, PMID: 32039048 PMC6987142

[ref33] Muri J. Kopf M. (2020). Redox regulation of immunometabolism. Nat. Rev. Immunol. 21, 363–381. doi: 10.1038/s41577-020-00478-8 33340021

[ref34] Mutak A. (2018). "cosinor2: extended tools for Cosinor analysis of rhythms. R package version 0.2.1". Available at: https://CRAN.R-project.org/package=cosinor2

[ref35] Noverr M. C. Huffnagle G. B. (2004). Regulation of *Candida albicans* morphogenesis by fatty acid metabolites. Infect. Immun. 72, 6206–6210. doi: 10.1128/IAI.72.11.6206-6210.2004, PMID: 15501745 PMC523025

[ref36] Oksanen J. Simpson G. Blanchet F. Kindt R. Legendre P. Minchin P.. (2022). "Vegan: Community ecology package. R package version 2.6-4". Available at: https://CRAN.R-project.org/package=vegan

[ref37] Pan L. L. Niu W. Fang X. Liang W. Li H. Chen W.. (2019). *Clostridium butyricum* strains suppress experimental acute pancreatitis by maintaining intestinal homeostasis. Mol. Nutr. Food Res. 63:e1801419. doi: 10.1002/mnfr.201801419, PMID: 31034143

[ref38] Panda S. (2016). Circadian physiology of metabolism. Science 354, 1008–1015. doi: 10.1126/science.aah4967, PMID: 27885007 PMC7261592

[ref39] Park M. Yoo J. H. Lee Y. S. Park E. J. Lee H. J. (2020). Ameliorative effects of black ginseng on nonalcoholic fatty liver disease in free fatty acid-induced HepG2 cells and high-fat/high-fructose diet-fed mice. J. Ginseng Res. 44, 350–361. doi: 10.1016/j.jgr.2019.09.004, PMID: 32148418 PMC7031749

[ref40] Pataky Z. Genton L. Spahr L. Lazarevic V. Terraz S. Gaia N.. (2016). Impact of hypocaloric Hyperproteic diet on gut microbiota in overweight or obese patients with nonalcoholic fatty liver disease: a pilot study. Dig. Dis. Sci. 61, 2721–2731. doi: 10.1007/s10620-016-4179-1 27142672

[ref41] Rastogi D. (2020). Pediatric obesity-related asthma: a prototype of pediatric severe non-T2 asthma. Pediatr. Pulmonol. 55, 809–817. doi: 10.1002/ppul.24600, PMID: 31912992 PMC7694442

[ref42] Reitmeier S. Kiessling S. Clavel T. List M. Almeida E. L. Ghosh T. S.. (2020). Arrhythmic gut microbiome signatures predict risk of type 2 diabetes. Cell Host Microbe 28, 258–272.e6. doi: 10.1016/j.chom.2020.06.004, PMID: 32619440

[ref43] Roenneberg T. Merrow M. (2016). The circadian clock and human health. Curr. Biol. 26, R432–R443. doi: 10.1016/j.cub.2016.04.011 27218855

[ref44] Rohart F. G. B. Singh A. Le Cao K.-A. (2017). mixOmics: an R package for 'omics feature selection and multiple data integration. PLoS Comput. Biol. 13:e1005752. doi: 10.1371/journal.pcbi.1005752, PMID: 29099853 PMC5687754

[ref45] RR Core Team, (2022). "R: A language and environment for statistical computing". 4.2.1 ed. (Vienna, Austria: R Foundation for Statistical Computing).

[ref46] Safari Z. Gérard P. (2019). The links between the gut microbiome and non-alcoholic fatty liver disease (NAFLD). Cell. Mol. Life Sci. 76, 1541–1558. doi: 10.1007/s00018-019-03011-w 30683985 PMC11105223

[ref47] Saran A. R. Dave S. Zarrinpar A. (2020). Circadian rhythms in the pathogenesis and treatment of fatty liver disease. Gastroenterology 158, 1948–1966.e1. doi: 10.1053/j.gastro.2020.01.050, PMID: 32061597 PMC7279714

[ref48] Skalski J. H. Limon J. J. Sharma P. Gargus M. D. Nguyen C. Tang J.. (2018). Expansion of commensal fungus Wallemia mellicola in the gastrointestinal mycobiota enhances the severity of allergic airway disease in mice. PLoS Pathog. 14:e1007260. doi: 10.1371/journal.ppat.1007260, PMID: 30235351 PMC6147580

[ref49] Szóstak N. Figlerowicz M. Philips A. (2023). The emerging role of the gut mycobiome in liver diseases. Gut Microbes 15:2211922. doi: 10.1080/19490976.2023.2211922, PMID: 37184158 PMC10187078

[ref50] Thaiss C. A. Zeevi D. Levy M. Zilberman-Schapira G. Suez J. Tengeler A. C.. (2014). Transkingdom control of microbiota diurnal oscillations promotes metabolic homeostasis. Cells 159, 514–529. doi: 10.1016/j.cell.2014.09.048, PMID: 25417104

[ref51] van Tilburg Bernardes E. Pettersen V. K. Gutierrez M. W. Laforest-Lapointe I. Jendzjowsky N. G. Cavin J. B.. (2020). Intestinal fungi are causally implicated in microbiome assembly and immune development in mice. Nat. Commun. 11:2577. doi: 10.1038/s41467-020-16431-1, PMID: 32444671 PMC7244730

[ref52] Walker W. H. Walton J. C. DeVries A. C. Nelson R. J. (2020). Circadian rhythm disruption and mental health. Transl. Psychiatry 10. doi: 10.1038/s41398-020-0694-0, PMID: 32066704 PMC7026420

[ref53] Wang H. Zhang H. Su Y. (2022). New insights into the diurnal rhythmicity of gut microbiota and its crosstalk with host circadian rhythm. Animals 12:1677. doi: 10.3390/ani12131677, PMID: 35804575 PMC9264800

[ref54] Wei L. Yue F. Xing L. Wu S. Shi Y. Li J.. (2020). Constant light exposure alters gut microbiota and promotes the progression of steatohepatitis in high fat diet rats. Front. Microbiol. 11:1975. doi: 10.3389/fmicb.2020.01975, PMID: 32973715 PMC7472380

[ref55] Xue S.-J. Chi Z. Zhang Y. Li Y.-F. Liu G.-L. Jiang H.. (2018). Fatty acids from oleaginous yeasts and yeast-like fungi and their potential applications. Crit. Rev. Biotechnol. 38, 1049–1060. doi: 10.1080/07388551.2018.1428167, PMID: 29385857

[ref56] Xue L. Deng Z. Luo W. He X. Chen Y. (2022). Effect of Fecal microbiota transplantation on non-alcoholic fatty liver disease: a randomized clinical trial. Front. Cell. Infect. Microbiol. 12:759306. doi: 10.3389/fcimb.2022.759306, PMID: 35860380 PMC9289257

[ref57] Ye Y. Xu H. Xie Z. Wang L. Sun Y. Yang H.. (2020). Time-restricted feeding reduces the detrimental effects of a high-fat diet, possibly by modulating the circadian rhythm of hepatic lipid metabolism and gut microbiota. Front. Nutr. 7:596285. doi: 10.3389/fnut.2020.596285, PMID: 33425971 PMC7793950

[ref58] Yonatan Y. Amit G. Friedman J. Bashan A. (2022). Complexity-stability trade-off in empirical microbial ecosystems. Nat. Ecol. Evol. 6, 693–700. doi: 10.1038/s41559-022-01745-8, PMID: 35484221

[ref59] Younossi Z. Anstee Q. M. Marietti M. Hardy T. Henry L. Eslam M.. (2018). Global burden of NAFLD and NASH: trends, predictions, risk factors and prevention. Nat. Rev. Gastroenterol. Hepatol. 15, 11–20. doi: 10.1038/nrgastro.2017.109, PMID: 28930295

[ref60] Zarrinpar A. Chaix A. Yooseph S. Panda S. (2014). Diet and feeding pattern affect the diurnal dynamics of the gut microbiome. Cell Metab. 20, 1006–1017. doi: 10.1016/j.cmet.2014.11.008, PMID: 25470548 PMC4255146

[ref61] Zeng S. Schnabl B. (2022). Roles for the mycobiome in liver disease. Liver Int. 42, 729–741. doi: 10.1111/liv.15160, PMID: 34995410 PMC8930708

[ref62] Zhang F. Aschenbrenner D. Yoo J. Y. Zuo T. (2022). The gut mycobiome in health, disease, and clinical applications in association with the gut bacterial microbiome assembly. Lancet Microbe 3, e969–e983. doi: 10.1016/s2666-5247(22)00203-8, PMID: 36182668

[ref63] Zhang X. Wu M. Liu Z. Yuan H. Wu X. Shi T.. (2021). Increasing prevalence of NAFLD/NASH among children, adolescents and young adults from 1990 to 2017: a population-based observational study. BMJ Open 11:e042843. doi: 10.1136/bmjopen-2020-042843, PMID: 33947727 PMC8098935

[ref64] Zhu L. Baker R. D. Baker S. S. (2014). Gut microbiome and nonalcoholic fatty liver diseases. Pediatr. Res. 77, 245–251. doi: 10.1038/pr.2014.157 25310763

