# Supplementary material for: Chronic jet lag alters gut microbiome and mycobiome and promotes the progression of MAFLD in HFHFD-fed mice
Source: Front Microbiol. 2023 Dec 7;14:1295869. doi: 10.3389/fmicb.2023.1295869 (PMC10733492; doi:10.3389/fmicb.2023.1295869)
Supplement: Supplementary file 1 [file Table_1.DOCX]

Supplementary Material

# Supplementary Table

## Table S1 Primers used for qRT-PCR

| Gene | Forward primer (5’-3’) | Reverse primer (3’-5’) |
| --- | --- | --- |
| Clock | GAGGTCGTCCTTCAGCAGTC | TGTGACATGCCTTGTGGAAT |
| Bmal1 | ACAACGAGGGCTGCAACCT | CCCGTTCGCTGGTTGTG |
| Cry1 | AGGGAACCCCATCTGTGTTC | TGGTGCATTCCAAGGATCGT |
| Cry2 | ATGTGTTCCCAAGGCTGTTC | CCTCCTTGGCCATCTTCATA |
| Per1 | CAGCTGGGCCGGTTTTG | CACTTTATGGCGACCCAACA |
| Per2 | ATCTCCAGGCGGTGTTGAAG | AGGGTTACGTCTGGGCCTCT |
| GAPDH | GGATGCTGCCCTTACCC | GTTCACACCGACCTTCACC |
